# Does apical membrane GLUT2 have a role in intestinal glucose uptake?

**DOI:** 10.12688/f1000research.5934.1

**Published:** 2014-12-12

**Authors:** Richard J Naftalin

**Affiliations:** 1 Department of Physiology and BHF Centre of Research Excellence, King's College London, School of Medicine, London, SE1 9HN, UK

## Abstract

It has been proposed that the non-saturable component of intestinal glucose absorption, apparent following prolonged exposure to high intraluminal glucose concentrations, is mediated via the low affinity glucose and fructose transporter, GLUT2, upregulated within the small intestinal apical border.

The evidence that the non-saturable transport component is mediated via an apical membrane sugar transporter is that it is inhibited by phloretin, after exposure to phloridzin. Since the other apical membrane sugar transporter, GLUT5, is insensitive to inhibition by either cytochalasin B, or phloretin, GLUT2 was deduced to be the low affinity sugar transport route.

As in its uninhibited state, polarized intestinal glucose absorption depends both on coupled entry of glucose and sodium across the brush border membrane and on the enterocyte cytosolic glucose concentration exceeding that in both luminal and submucosal interstitial fluids, upregulation of GLUT2 within the intestinal brush border will usually stimulate downhill glucose reflux to the intestinal lumen from the enterocytes; thereby reducing, rather than enhancing net glucose absorption across the luminal surface.

These states are simulated with a computer model generating solutions to the differential equations for glucose, Na and water flows between luminal, cell, interstitial and capillary compartments. The model demonstrates that uphill glucose transport via SGLT1 into enterocytes, when short-circuited by any passive glucose carrier in the apical membrane, such as GLUT2, will reduce transcellular glucose absorption and thereby lead to increased paracellular flow. The model also illustrates that apical GLUT2 may usefully act as an osmoregulator to prevent excessive enterocyte volume change with altered luminal glucose concentrations.

## Introduction

Intestinal glucose absorption has been studied for more than a century and still remains controversial. During the last fifty years the main research thrust has been to identify and characterize the individual transport components within the intestinal epithelium. This progressively reductivist approach has been very successful: we have a comprehensive knowledge of the nature of the driving forces generating sugar absorption; the specificity range of the sugar transporters involved; their sites of activity within the enterocytes and of how the individual transport processes function at a molecular level ^1– 3^. Less clear is how the intestine functions as a working ensemble to absorb glucose over the wide range of luminal concentrations occurring within the small intestine and how this process is controlled, both in the short and long-term. These uncertainties arise from the multiplicity and complexity of interactive processes and lack of a comprehensive model permitting an integrated view of intestinal glucose uptake.

**Figure 1.**
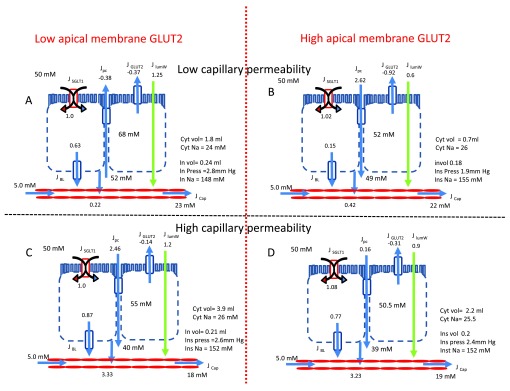
These diagrams show snapshots of the simulated glucose flows from intestinal lumen loaded with 50 mM in 150 mM NaCl to capillaries perfusing the submucosal spaces, whose afferent arterial concentration contains 150 mM NaCl and 5 mM D glucose. The tissue in panel **A** has low apical GLUT2 and GLUT5 activity and low capillary permeability and perfusion rates (clearance). In panel **B** the tissue apical membrane GLUT2 activity is increased by 4-fold above that in panel **A**, capillary perfusion is unchanged. In panel **C**, the apical GLUT2 activity is the same as in panel **A**, but capillary clearance is increased by 10-fold. In panel **D**, the apical GLUT2 is raised, as in panel **B** and the capillary clearance raised, as in panel **C**. The rates of glucose uptake are normalized relative to the rate of SGLT1 glucose uptake (panel **A**). Altering either GLUT2, or capillary clearance have negligible effects on glucose inflow via SGLT1. However, after raising the apical GLUT2 activity, the steady state glucose concentration within the cytosol decreases from 68 to 52 mM (c.f. Panels **A** and **C**). On raising capillary clearance, the steady state of cytosolic glucose concentration also decreases (c.f. Panel **A** versus Panel **C** and Panel **B** versus Panel **D**). Raising capillary glucose clearance increases the rate of glucose inflow from the interstitial to capillary fluid by fourteen fold (c.f. Panel **A** and **C**). These changes are accompanied by decreased interstitial fluid glucose from 52 to 40 mM and reductions in the mean capillary glucose from 23 to 18 mM. Reduced interstitial glucose concentrations reverse the direction of the glucose gradient across the paracellular pathway from -2 to + 10 mM. Thus raising the capillary clearance of glucose, reverses the direction of paracellular glucose flow from (-0.38) to (+2.46) and increases the net glucose inflow across the luminal surface from (0.22 to 3.23). Although raising apical membrane GLUT2 activity by fourfold reduces net glucose influx across the apical border from 0.63 to 0.15, it also indirectly leads to an increase in paracellular glucose flux and thereby causes a slight increase in net glucose flux across the luminal border. When capillary clearance is raised, either by enhanced perfusion rates, or increased endothelial permeability, increasing apical membrane GLUT2 enhances apical membrane glucose reflux from -0.14 to -0.31. This has no significant effect on glucose flow from the interstitial to capillary fluid. (c.f. panel **C** and **D**).

The early opinion on intestinal glucose transport was that stereospecific electrogenic active transcellular transport process coexisted with a variable non-specific paracellular diffusive flux ^4– 8^. Intestinal glucose absorption entails specific sodium-dependent hexose interactions with jejunal and ileal enterocyte glucose transporters in the apical and sodium-independent passive downhill transport via basal-lateral membranes and transit by solvent drag via non-selective paracellular pathways, generated by electro-osmotic flow of Na ^+^ and water ^7, 9, 10^, or by paracellular passive diffusion down the glucose concentration gradient existing between the intestinal lumen and lamina propria ^11, 12^. This diffusive route permits non-specific transport of L-glucose, D-rhamnose, or mannitol, as well as D-glucose at rates that are correlated with net fluid transport ^13^. The general consensus was that at around a luminal glucose ≈ 25 mM the active and passive components are about equal and above this passive absorption becomes dominant ( Figure 1).

This dual transport model explained why the apparent affinity of total net glucose uptake is much less, K _m_ > 62.3±3.2 mM than the K _m_ obtained for electrogenic glucose transport (K _m_ = 17.9±0.4 mM); and why phloridzin, a blocker of Na-coupled glucose transport via SGLT1 at the luminal surface, affects mainly electrogenic transport, but not transport via the paracellular route ^4^.

Parsons and colleagues ^14, 15^ were amongst the first to postulate parallel active and passive absorptive processes in the luminal surface intestinal membrane.

Kellett and colleagues ^1, 16, 17^ later proposed that when luminal glucose is raised above 15 mM, that the non-saturable absorptive component, instead of being via the paracellular route is due to influx via a low affinity glucose transporter, GLUT2, whose presence is regulated within jejunal and ileal enterocytes apical membranes. The salient experimental evidence supporting this view is that the “non-saturable” component of glucose absorption is inhibited by either high phloretin (0.75–1 mM), or high cytochalasin B (0.2 mM) concentrations, both of which inhibit GLUT2 and neither of which inhibit GLUT5.

Using a sigmoid curve fit, Kellett and Helliwell ^1^ obtained a K _m_ of the phloretin-sensitive component “similar” to that of GLUT2, 56±14 mM; n=1.6±0.4. They argued that GLUT2 is the most likely route for this low affinity transport, since it also transports fructose. Later reports showed that artificial sweeteners e.g. aspartame, sucralose and saccharin in parallel with an increase in intracellular calcium, increase the rate of glucose absorption, by increasing brush border GLUT2 ^18^ and this in turn increased release of several incretins gluco-insulinotropic peptide(GIP); glucagon- like peptide (GLP-1) and peptide tyrosine-tyrosine (PYY) from enteroendocrine cells ^19^.

Although these arguments seem plausible, there are several reasons to question the assertion that apical membrane GLUT2 mediates the low affinity component of intestinal D-glucose absorption. Many studies have shown that the low affinity glucose absorptive route has low specificity- it can transport sugars e.g. L-glucose or rhamnose, or low molecular weight solutes, such as Cr-EDTA, or mannitol, that are not transported by any GLUTs ^13^. Thus the explanation that GLUT2 is the sole mediator of the low affinity sugar transport route does not explain transport of these paracellular markers without any affinity for sugar transporters.

The K _m_ of GLUT2 has been measured as approximately 17 mM ^20, 21^, this value is much lower than the very high K _m_ 56±14 mM observed by Kellett & Helliwell (2000) ^1^. Additionally, at luminal glucose concentrations > 50 mM absorption linearly correlates with luminal concentration; i.e. is not saturable ^8^. Thus the high K _m_ of the “phloretin-sensitive” component does not necessarily signify glucose transport via a low affinity glucose transporter.

Furthermore, phloretin- is not uniquely specific as a glucose-transporter inhibitor. Phloretin also blocks chloride, or aquaporin water channels, or urea transporter mediated urea and water transport, probably by intercalating with the lipid membrane and consequently may also inhibit solute and water paracellular transport ^22, 23^. Hence, a transport process blocked by high concentrations of phloretin or cytochalasin B need not imply that the inhibited flow is mediated via apical membrane GLUT2.

In contrast to Kellett and colleagues’ claims, other studies with GLUT2 knock out (KO) mice have shown that GLUT2 makes no substantial contribution to net glucose absorption and furthermore that D-glucose accumulation in enterocytes is increased in GLUT2 KO mice ^20, 24^. This increase can in part be ascribed to loss of GLUT2 mediated transport activity from the baso-lateral membranes. Doubts have also been raised as to whether GLUT2 is expressed at all in the intestinal apical membranes ^25^. Roder *et al.* ^24^, were unable to detect significant levels of GLUT2 within the intestinal brush borders of wild type mice. Additionally, in humans there is an absence of any detectible increased response to artificial sweeteners with relation to any increased sugar uptake, or incretin release ^26, 27^.

However, Kellett ^28^, has responded to some of these arguments, suggesting that the mice used in these KO studies were not optimally prepared. Starvation leads to loss of both intestinal GLUT2 apical protein and GLUT2 mRNA, whereas re-feeding after a period of starvation leads to a rapid increase in both apical GLUT2 expression and to GLUT2 mRNA expression within the intestine ^29^.

The later results reported by Brot-Laroche’s group appear to conflict with some earlier data from her laboratory showing that semi-starvation increased the V _max_ and K _m_ of D-glucose uptake into guinea pig jejunal brush border membrane vesicles (BBMV) ^30^. Starvation was postulated to induce a secondary low affinity glucose transport system. Additional studies revealed that phloretin (0.25 mM) enhanced the initial rate of D-glucose uptake by 15% into guinea-pig BBMV. Application of Student’s two-tailed t–test shows that this increase is significant (P < 0.012). Cytochalasin B (0.1 mM) inhibited D-glucose (10 mM) uptake by 38% (p < 0.0001), but had negligible effects on SGLT1 specific α-methyl-D-glucoside uptake ^31^. The earlier results imply that phloretin enhances, rather than inhibits, the low affinity D-glucose transport in BBMV, as was later asserted ^1, 18^.

Recent live imaging studies indicate that GLUT2 is a variable presence within the apical membrane ^32^; its trafficking being dependent on signals induced by high intracellular glucose concentrations.

## Analysis

Although upregulation of apical membrane GLUT2 is a feature of raised luminal D-glucose concentrations, it is far from clear, as contended, that this leads to enhanced net glucose transport ^1, 17^. Since both active (SGLT1) and passive glucose transporter (GLUTs 2 and 5) elements are present within the brush border membranes, the kinetics of net glucose flow across the brush border ensemble will depend both on the variable glucose concentrations in the adjacent luminal and cytosolic compartments and the relative proportions of active and passive transport components and the area of absorbing intestinal surface exposed to glucose. The steady state cytosolic and interstitial glucose concentrations are also reliant upon the concentration dependence of flows across the baso-lateral membrane into the interstitial fluid and between the luminal fluid and interstitial fluid via the intercellular junctions. GLUT2 (K _m_ ≈ 17 mM) is the main transporter for glucose movement across the basal-lateral membranes ^33, 34^.

The apparent transport parameters (K _m_ and V _max_) ^4– 6^ obtained. In intestinal enterocytes *in situ*, where flows with varying luminal glucose concentrations are normally measured in steady state, have scant resemblance to those obtained in *zero-trans* conditions with isolated membrane vesicles or oocytes.

Uphill glucose transport via the apical membrane sodium-glucose cotransporter SGLT1 generates polarized sugar flow, causing the intracellular glucose concentration to increase: eventually the cytosolic and also interstitial glucose concentrations may exceed the luminal concentration ^35^. Once these conditions are met, glucose will reflux back into the intestinal lumen via passive transporters in the apical membrane, or via the tight junction ( Figure 1A). If the V _max_ of the passive apical membrane glucose transporters is raised, then owing to enhanced glucose reflux via GLUT2, net glucose influx across the apical membrane will be reduced. However, net glucose uptake across the luminal surface, including the paracellular pathways may be augmented. This increase in paracellular glucose flow arises from decreased transcellular flow. The resulting slight decrease in interstitial fluid glucose concentration increases the gradient between the intestinal lumen and interstitial fluid, ( Figure 1B) and Figure 3(A–C).

Glucose influx across the apical membrane remains polarized over a very wide concentration range, due to the very low affinity for glucose at the export site of SGLT1 ^35, 36^. Thus when both the cytosolic and the interstitial glucose concentrations are close to GLUT2 saturation levels; i.e. D-glucose > 30 mM, the resistance to glucose outflow across the baso-lateral membrane will increase. Consequently, cytosolic concentration may increase disproportionally as luminal glucose concentrations rise, since the apical and baso-lateral membranes may act as a double membrane rectifier to glucose flow ^37^. This promotes non-linear glucose accumulations in the intermediate cytosol between the apical and baso-lateral membranes ^11, 38, 39^ ( Figure 1A).

Glucose flow from the interstitial fluid into the villus capillaries depends on the glucose diffusion between the interstitial fluid and the mean capillary luminal concentration. The mean capillary luminal glucose concentration is a complex non-linear function of the glucose permeability of the capillary membranes, the systemic arterial glucose concentration and the capillary flow rate ^11^. The boundary conditions of this flow network determine the steady-state glucose concentrations within all the intermediate compartments.

Raising intestinal luminal glucose above 30 mM results in increased superior mesenteric arterial flow from around 1000–2500 ml min ^-1^ ^40– 42^. Raised capillary glucose clearance will reduce the interstitial glucose concentration, thereby also reducing cytosolic glucose concentration, thus increasing net influx across the luminal surface, whilst reducing glucose reflux both via brush border passive transporters and via the paracellular pathway. The model simulates all these conditions as seen by comparing Figures 1A and 1B with Figures 1C and 1D.

**Figure 2.**
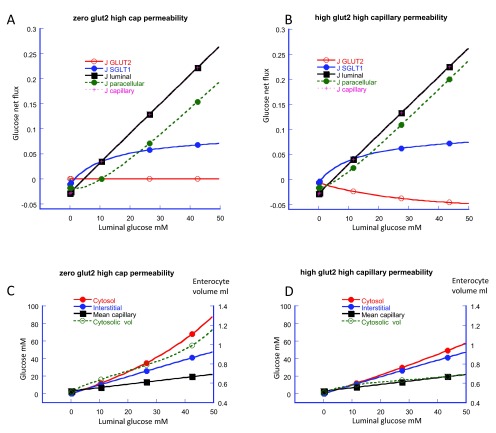
Simulation of altered apical membrane GLUT2 activity on intestinal glucose fluxes from zero (Panel **A**) to an arbitrary of maximal flux value of 2 (Panel **B**). The simulations show the glucose fluxes via apical SGLT1 (blue); apical GLUT2 (red); paracellular pathway (green); the total transluminal membrane, (SGLT1 + GLUT2 + paracellular fluxes), (black) and interstitial to capillary flow (pink crosses) inset on the black square. The main effect of increasing GLUT2 is to cause a negative glucose flux (backflux) via GLUT2 (Panel **B**). This is accompanied by a increased paracellular flux without any significant change in net transluminal or transepithelial glucose flux. The point at which paracellular glucose flux and SGLT1 flux are equal lies between 20 and 30 mM as has been previously observed ^4– 6^. This value is used as one of the key registration points for the model. The cytosolic (red) interstitial (blue) and mean capillary glucose concentrations (black) and enterocyte volume per unit weight of tissue (green) are shown in panel **C** with zero apical GLUT2 and in Panel **D** with GLUT2 V _max_ = 2. Increased apical GLUT2 activity decreases cytosolic glucose concentration (panel **D**). With rising luminal glucose concentration raised GLUT2 activity prevents the non-linear increase in enterocyte volume seen with zero GLUT2 (Panel **C**).

However, even with high rates of vascular perfusion, the interstitial glucose concentration approximates to that of the luminal concentration. Consequently, as luminal glucose concentrations are raised, even although interstitial capillary glucose clearance is increased, the enterocyte cytosolic concentrations continuously rises, ( Figure 2C and 2D) ^11^.

**Figure 3.**
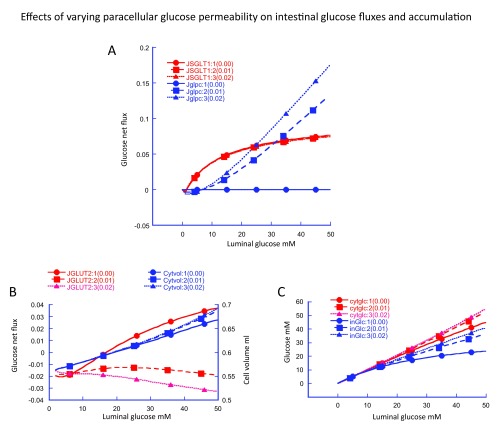
Simulation of effects of varying paracellular glucose permeability on intestinal glucose fluxes and accumulation. The effects of varying paracellular glucose permeability P _glc_ from 0 to 0.02 cm s ^-1^ are shown in Figure 3A increasing P _glc_ on paracellular glucose flux (Blue) .As P _glc_ is increased from zero the point of equality of paracellular glucose flux Jglpc with glucose flux via SGLT1 decreases from infinity at P _glc_ = 0 to around 20–30 mM luminal glucose when P _glc_ = 0.01–0.02 cm s ^-1^. Increases P _glc_ raises interstitial glucose concentrations 3C (blue) and in parallel, cysosolic concentrations Figure 3C (red). The reduction in glucose gradient across the basolateral membrane with raised P _glc_ reduces and then reverses glucose flux via GLUT2 ( Figure 3B).

Capillary clearance of glucose is a key factor affecting net intestinal glucose absorption at the intestinal border, as submucosal capillary glucose concentration rapidly equilibrates with that in the interstitial solution ^15^. Intestinal glucose clearance depends on the local blood flow rate, determined by the superior mesenteric arterial (SMA) pressure and its compliance and also the mean glucose concentration difference between the villus capillaries and the interstitial solution ( Figure 1A–D). Thus, as can be seen by comparing Figures 1A and 1B with Figures 1C and 1D, increasing the capillary clearance reduces the interstitial glucose concentration, thereby increasing the glucose gradients across the baso-lateral membranes and between the luminal and interstitial solutions, thereby enhancing absorptive flux.

With constant high capillary glucose clearance, increasing apical GLUT2 activity, whilst enhancing glucose backflux across the apical membrane, also increases paracellular absorption. This tends to nullify the GLUT2-induced decrease in apical membrane net absorption.

Additional complexity is introduced by glucose-coupled Na ^+^ and water flows altering cytosolic and interstitial osmolarities, thereby generating changes in enterocyte cytosolic and interstitial fluid volume and interstitial pressure. The interstitial pressure changes affect fluid and solute flows via the paracellular pathway and via the capillaries and lymphatics ^43^. The effects on water flows are shown with green arrows in Figures 1A–D. As modelled here, changing the maximal rate of apical GLUT2 or capillary perfusion rates have relatively smaller effects on net water than on glucose flows.

This is also illustrated in Figures 2A and B, where increasing apical GLUT2 activity from zero ( Figure 2A) to a high level ( Figure 2B), increases GLUT2 backflux and also enhances glucose influx via the paracellular route. Consequently the net effect of altering apical GLUT2 activity on luminal glucose absorption is almost zero.

### Effects of varying paracellular glucose permeability

Increased paracellular glucose diffusion has multiple effects on glucose fluxes and accumulation. Increasing paracellular glucose permeability directly increases paracellular glucose flux ( Figure 3A (blue)). This increases the interstitial glucose concentration ( Figure 3C (blue)). Raising interstitial glucose concentration decreases the glucose concentration gradient across the basolateral membrane, thereby decreasing basolateral glucose flux and raising cytosolic glucose concentration ( Figure 3C (red)). Increasing cytosolic glucose concentration reverses the direction of glucose flow across the apical membrane via GLUT2 ( Figure 3B (red)), but is without significant effect on glucose flux via SGLT1 ( Figure 3A), or cell volume ( Figure 3B (blue)).

Thus, it is evident that paracellular glucose diffusion significantly alters glucose fluxes, both directly via the paracellular and indirectly on the passive glucose fluxes at the apical and baso-lateral membranes. Only when paracellular glucose flux is close to zero is there any significant glucose influx via GLUT2 ( Figure 3B).

### Effects of phloridzin and phloretin on intestinal glucose transport

The effect of phloridzin is to block SGLT1 without affecting GLUT2 ^1^. As previously discussed, phloretin inhibits GLUTs 1-IV, but not GLUT5. However, it has additional effects on chloride, urea and water permeability, so also affects paracellular conductivity ^22, 23^.

**Figure 4.**
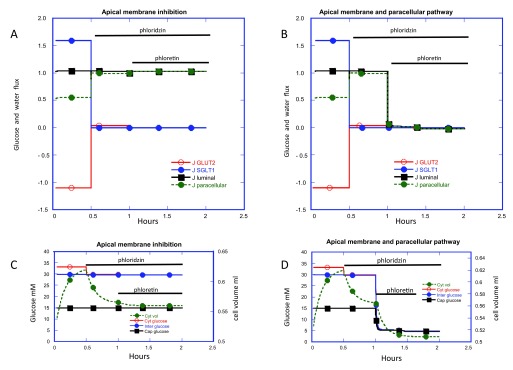
Simulation of sequential additions of phloridzin then phloretin to the luminal fluid on intestinal glucose fluxes. GLUT2 is present in the apical membrane V _max_ 2 and the capillary perfusion rate = 10 is similar to that shown in Figure 2 panels **B** and **D**. The luminal glucose is 30 mM and afferent capillary glucose is 5 mM to simulate the conditions used by Kellett & Helliwell 2000 ^1^. In Figure 4, panels **A** and **B** inhibition of SGLT1 activity at 0.5h to zero reduces glucose flux via SGLT1 to zero. Simultaneously glucose flux via GLUT2 increases thereby reversing the backflux from -1.1. to 0.05 and also paracellular glucose flux increases from 0.55 to 0.95. In panels **C** and **D** the glucose concentration changes in the cytosol (red) interstitial fluid (blue) capillary fluid (black) and cytosolic volume (green). Following phloridzin addition and inhibition of SGLT1, cytosolic glucose falls from 33 to 30 mM; cytosolic volume falls from 0.62 to 0.56 ml at 1 hour without significant changes in capillary or interstitial fluid glucose concentration. This explains both the fall in glucose reflux via GLUT2 and the decrease in basolateral membrane flux is compensated by the rise in paracellular flux thereby nullifying interstitial glucose concentration changes. Phloretin addition at 1h is simulated by blocking apical GLUT2 (panel **A**) and by blocking both apical GLUT2 and paracellular glucose and Na permeability (panel **B**). GLUT2 fluxes fall to zero in both panels **A** and **B** and in panel **A** there is a small increase in paracellular glucose flow but the total transluminal glucose flux is unaffected by addition of phloretin after phloridzin. There is a small decrease in cytosolic volume from 0.56 to ≈ 0.55. the paracellular flux falls to zero as does the transluminal glucose flux in panel **B**, simulating the effect observed by Kellett and Helliwell 2000 ^1^. This is accompanied by a large decrease in cell volume from 0.56 to 0.51 ml. Since no net glucose transport now occurs from the luminal fluid, capillary glucose concentration also decreases to 5 mM.

Simulation of the temporal effects of phloridzin on intestinal glucose uptake exposed to luminal glucose 30 mM ^1^ shows that whilst inhibiting glucose influx via SGLT1, net glucose efflux via GLUT2 is abolished as a result of the decreased uphill glucose accumulation in the cytosol. Hence glucose flux across the basolateral membrane is reduced; however, because the interstitial glucose decreases due to diminished, transcellular flow paracellular glucose influx rises. Consequently, the net effect of SGLT1 inhibition by phloridzin on net glucose absorption is negligible, as observed in rabbit ileum pre-incubated with glucose ^9^. Following phloridzin inhibition of SGLT1, cytosolic glucose falls from ≈ 32 mM to ≈ 17 mM as simulated here ( Figure 4A and 4B).

Subsequent inhibition of apical GLUT2 by phloretin is accompanied only by a very small decrease in net glucose influx as it falls to zero. However, this decrease in net transcellular glucose influx is supplemented by a reciprocal increase in paracellular flux, so that there is still a negligible change in net luminal glucose absorption. Thus, when glucose fluxes via apical SGLT1 and GLUT2 are completely inhibited, only the paracellular route remains to permit luminal to submucosal glucose flow and this flux rises to compensate for the reduced transcellular flow as a result of reduced interstitial glucose concentration.

The simulation shows that if phloretin inhibits only apical GLUT2, then it exerts no significant effect on luminal glucose uptake Figure 4A. If instead of only inhibiting glucose flux via apical GLUT2, phloretin also inhibits paracellular glucose and electrolyte fluxes, then the observed effect on intestinal glucose absorption ( Figure 4B) is similar to that observed by Kellett and Helliwell ^1^; namely, reduction in net luminal glucose flux to zero. The cytosolic glucose together with the interstitial glucose concentrations now fall to 5 mM; equal to the sink capillary glucose concentration, since now there is no compensatory rise in paracellular glucose flux occurring when interstitial glucose concentration is reduced.

### Effects of GLUT2 on enterocyte volume during glucose loading via SGLT1

GLUT2 functions as an apical glucose shunt, thereby reducing cytosolic glucose accumulation by SGLT1. This shunt functions in two important ways, first by reducing net luminal influx, rather than increasing it as previously deduced ^1^. It will also redistribute the luminal glucose to more distal intestinal regions, consequently exposing larger intestinal surface areas to luminal glucose.

**Figure 5.**
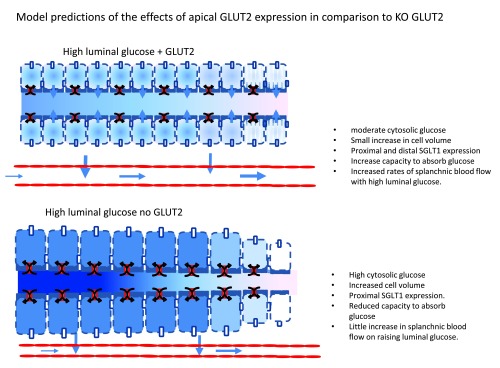
Diagram showing the predicted effects from the simulation model of loading enterocytes with luminal fluid containing 50 mM glucose and 5 mM glucose in the capillary perfusion fluid in A normal enterocytes expressing apical membrane GLUT2 and in B GLUT2 KO enterocytes. The KO cells have higher enterocyte glucose concentrations in the proximal intestine, but higher paracellular flow and larger cell volumes. The normal enterocytes have lower cytosolic glucose concentrations and SGLT1 is more widely dispersed along the intestinal length with higher rates of glucose permeation in distal regions of the small intestine. Long term exposure may lead to higher maximal glucose absorption rates in normal intestine than with GLUT2 KO.

This latter effect may explain why when pigs are exposed to high carbohydrate diets raised SGLT1 protein and mRNA expression is observed in more distal intestinal regions ^44^. More SGLT1 is also observed in duodenal epithelia of morbidly obese humans ^45^. Increased density and increased area of intestinal SGLT1 expression implies that the intestine develops the capacity to deal with increased carbohydrate loads by absorbing more carbohydrate in aggregate, although not per unit area ( Figure 5). This will generate higher concentration peaks of carbohydrate in the splanchnic circulation following absorbable carbohydrate ingestion ^41, 45^ and higher rates of splanchnic blood flow in conscious animals ^46– 48^.

It would seem more likely that instead of a means of enhancing apical glucose absorption, GLUT2 behaves primarily as an osmoregulator to maintain enterocyte volume in the face of large and rapid changes in the luminal and cytosolic osmotic pressure following ingestion of carbohydrates, or their subsequent dilution upon drinking water.

Since glucose is one of the most variable osmolytes within the intestine and splanchnic circulation, it is likely that a rapid adaptation to hyper or hypo osmotic changes within the intestinal lumen via a GLUT2 shunt pathway in the apical membrane would provide a useful means of regulating enterocyte volume, thereby avoiding excessive membrane stress and cytolysis. The reduced local net influx would also result in redistribution of hypertonic luminal glucose to more distal regions where this excess glucose would be absorbed by SGLT1.

GLUT2 has not previously been considered as an osmoregulator of enterocyte volume. This role has been mainly assigned to potassium and chloride channels ^49^. Whilst ion channels certainly provide an important role in cell volume regulation, they may not be as well adapted as GLUT2 to fulfilling the enterocytes’ specialized needs for osmotic control due to large changes in sugar dependent osmotic gradients.

Simulation shows that glucose accumulation within the cytosol via SGLT1 is accompanied by an increased cytosolic volume Figures 2C and 2D, Figures 4C and 4D. The effect of increased rates of apical GLUT2 which prevents excessive glucose accumulation at high luminal glucose concentrations, compare Figure 2C with Figure 2D, also reduces enterocyte volume increase.

## Summary and conclusions

Kellett & Helliwell (2000) ^1^ have proposed that the non-saturable component of intestinal glucose absorption, apparent when luminal glucose is raised above 15 mM, is due to enhanced flux via the low affinity glucose transporter GLUT2, which they and others have observed ^32^ is present within the apical border of the jejunum and ileum following prolonged exposure to high intraluminal glucose or following activation of protein kinase C by phorbol myristate acetate.

Evidence in support of this contention is that this “non-saturable” component is inhibited by high phloretin or high cytochalasin B concentrations – which both can inhibit GLUT2. The K _m_ of the phloretin sensitive component claimed to be similar to that of GLUT2 approximately 56±14; n = 1.6±0.4.

They argue that GLUT2 is the most likely route for this low affinity transport, since it also transports fructose and the only other fructose transporter GLUT5 is insensitive to inhibition by either cytochalasin B or phloretin.

However, it is unclear that upregulation of GLUT2 within the intestinal brush border actually does enhance D-glucose absorption. At raised luminal glucose concentrations the cytosolic concentrations and the submucosal interstitial fluid glucose concentrations will exceed the intestinal luminal glucose concentrations, so GLUT2 will stimulate passive downhill glucose reflux from the enterocyte cytosol, thus reducing net glucose uptake across the luminal surface. This glucose backflux may be augmented by glucose reflux via the paracellular pathway when the interstitial glucose is raised. This will occur when the splanchnic capillary glucose concentration is raised above 10 mM, as occurs during ingestion of high glucose loads, or in hyperglycaemic states.

These states are simulated here with a model of intestinal glucose transport incorporating glucose sodium and water cotransport across the luminal border variable rates of apical GLUT2 and paracellular flows and variable rates of capillary clearance of solutes and water from the submucosal interstitial fluid.

The model demonstrates that apical membrane GLUT2 may usefully function as osmoregulator to prevent excessive enterocyte volume changes during glucose loading, or following sudden decreases in luminal glucose concentration.

## Methods

### Summary of model equations

The simultaneous flows of glucose Na and water from lumen across the apical membrane to cytosol and across the intercellular junctions from lumen to interstitial space followed by flows across the basolateral membrane of glucose Na and water to the interstitial space and from the interstitial space to the capillary lumen are modelled using Berkeley Madonna version 9.0119 http://www.berkeleymadonna.com/. Water flows generated by the osmotic pressure generated across the membrane boundaries between adjacent compartments generate volume changes in the cytosol and interstitial compartments. These volume changes are controlled by independent apical and baso-lateral hydraulic coefficients. Additionally, Na ^+^ and glucose flow via SGLT1 and GLUT2 are assumed to generate a coupled water flow ^50, 51^ and modelled as an additional component of water flux across both apical and basolateral membranes. The interstitial fluid compartment is assumed to have a non-linear elasticity similar to that observed by Granger ^52^ so that interstitial pressure rises non-linearly with volume.

The Na glucose and coupled flows and uncoupled flows via SGLT1 are modelled as outlined in ^53^ glucose flow across both apical and basolateral GLUT2 is modelled according to a simple two site model

i.e J _glucose_ = (G _out_/(1+G _out_)-G _in_/(1+G _in_).V _max_;

where G _out_ and G _in_ are the glucose concentration/K _m_(GLUT2) in the adjoining membrane compartments and K _m_ GLUT2 is the assigned GLUT2 K _m_ = 17 mM. Na flux basal-lateral membranes is assumed to have a similar kinetics between two to saturable sites to that of glucose K _m Na_ = 25 mM.

Additionally Na is pumped from the cytosol into the interstitial solution, according to the simple saturation equation

J _Napump_ = Na _cyt_.V _m(Napump)_/(Na _cyt_+K _m(Napump)_).

Flows of glucose and Na between the interstitial fluid and capillary fluid are assume to take the form of the convective diffusion equation

J _i_ = J _w_(C _o_+C _i_)/2 + P _i_(C _o_-C _i_).

Where J _i_ is the solute flux and J _w_ is water flux between interstitial fluid and capillary fluid C _o_ and C _i_ are the external and internal concentrations of solute _i_ and P _i_ is the permeability of solute _i_.

The water flow across the paracellular pathway Jwc _pc_ is determined by the osmotic and hydraulic pressure difference between the luminal and interstitial solutions hence Jwc _pc_ = L _p_ (2(Na _in_-Na _lum_).σ _Na_ + ((G _in_-G _lum_).σ _Glc_ - P _in_) where i _in_ and i _lum_ are the osmotic pressure of solutes I in the luminal and interstitial fluids σ _i_ is the reflection coefficient of solute I and P _in_ the interstitial pressure mm Hg.

## Appendix *{comments are italicised with curly brackets}*

  d/dt (Cytgl)= + Japgl – JGlbl *{ Cytgl = cytosolic glucose mM Japgl = glucose flux across the apical membrane = SGLT1+ GLUT2 flux; JGlbl = basolateral glucose flux.}*

     INIT Cytgl = 0 /Cytvol *{ INIT = initial ; cytvol = cytosolic volume /cm ^2^ }*

     LIMIT Cytgl >= 0

  d/dt (CytN)} = + JNap – JNbl *{CytN = cytosol Na mM; JNap = Na flux across apical membrane via SGLT1 coupled and uncoupled; JNbl = Na flux across baso-lateral membrane = Na pump flux and passive Na flux}.*

     INIT CytN = 1.5/Cytvol

     LIMIT CytN >= 0

  d/dt (inGlc) = + Jglpc - JcapGl + *JGlbl { Jglpc = paracellular glucose flux; JcapGL= glucose flux from interstitial to capillary fluid;}*

     INIT inGlc = 0/Invol *{inGlc = interstitial glucose concentration}*

  d/dt(InNc) = - JNcap + JNbl + JNpc { JNcap *{inNc= interstitial Na concentration; JNcap = Naflux from interstitial to capillary fluid; JNbl Na flux across basolateral membrane}*

     INIT InNc = 0/{Invol}

     LIMIT InNc >= 0

  d/dt(Invol)= + Jblw - Jcapw + Jpcw *{Invol = interstitial volume; Jblw = water flux across basolateral membrane; Jcapw= water flux between interstitial fluid and capillary fluid; Jpcw = paracellular water flux}*

     INIT Invol = *0.1 {invol= interstitial volume ml}*

     LIMIT Invol >= 0

  d/dt (Cytvol) = - Jblw + Japw *{Cytvol = cytosolic water volume; Jblw water flow across basolateral membrane includes osmotic and sugar coupled flows; Japw= waterflow across apical membrane includes osmotic and sugar coupled flows.}*

     INIT Cytvol = 0.5

     LIMIT Cytvol >= 0

## Berkeley Madonna equations

  {Reservoirs}

  d/dt (Cytgl) = + Japgl - JGlbl

     INIT Cytgl = 0 /Cytvol

     LIMIT Cytgl >= 0

  d/dt (CytN) = + JNap - JNbl

     INIT CytN = 1.5/Cytvol

     LIMIT CytN >= 0

  d/dt (inGlc) = + Jglpc - JcapGl + JGlbl

     INIT inGlc = 0/Invol

  d/dt (InNc) = - JNcap + JNbl + JNpc

     INIT InNc = 0/(Invol)

     LIMIT InNc >= 0

  d/dt (Invol) = + Jblw - Jcapw + Jpcw

     INIT Invol = 0.1

     LIMIT Invol >= 0

  d/dt (Cytvol) = - Jblw + Japw

     INIT Cytvol = 0.5

     LIMIT Cytvol >= 0

  {Flows}

  Japgl = (LumGl1sat-cyGLu1sat)*LGg+Lgn2*(LumNsat-CyNsat)+(LumGl2sat-Cyglu2sat)*VmGlap

  JGlbl = (cytglc/(cytglc+KmGbl)-InsGlc/(InsGlc+KmGbl))*VmGlbl

  JNap = (LumNsat-CyNsat)*LNn+(LumGl1sat-cyGLu1sat)*Lgn2

  JNbl = (CyNsat-InNsat)*LNn+Vpump*CyNsat

  JcapGl = Jcapw*(InsGlc+capGlc)/2+(InsGlc-capGlc)*PcapGl/1000

  JNcap = Jcapw*(InNc+capNc)/2+PcapN*(InNc-150)/1000

  Jglpc = (LumGlc-InsGlc)*Pglpc+Jpcw*(1-sigGl)*(LumGlc+InsGlc)/2

  JNpc = (LumN-InNc)*PNpc+Jpcw*(1-SigN)*(LumN+InNc)/2

  Jpcw = (insosm2-lumosm2+IntPressure)*(Lppc)/1E6

  Jcapw = IntPressure^2*Lpcap/1E6

  Japw = (cytosm-Lumosm)*Lpap/1E9+Japgl*Gwcoef/100

  Jblw = (Inosm-cytosm)*Lpbl /1E9

  {Functions}

  LumGl1nor = LumGlc/ KmGl1

  CyNnor = CytN/KmNap

  LumGlc = if time > 5000 then (time-5000)*1E-5 else 0

  LumN = 150

  LumNnor = LumN/KmNap

  LumGl1sat = LumGl1nor/(LumGl1nor+1)

  LumNsat = LumNnor/(LumNnor+1)

  LumGlc1nor = cytglc/KmGl1

  cyGLu1sat = LumGlc1nor/(LumGlc1nor+1)

  CyNsat = CyNnor/(CyNnor+1)

  InsGlc = inGlc

  Lumgl2nor = LumGlc/KmGl2

  LumGl2sat = Lumgl2nor/(Lumgl2nor+1)

  Cyglcnor = cytglc/KmGl2

  Cyglu2sat = Cyglcnor/(Cyglcnor+1)

  InNsat = InNc/KmNap/(InNc/KmNap+1)

  Lumosm = LumN*2+LumGlc

  Inosm = InNc*2*SigN+InsGlc*sigGl

  cytglc = Cytgl

  CyNc = CytN

  cytosm = fixosm+CyNc*2+cytglc

  fixosm = 125/Cytvol

  JNlum = JNap+JNpc

  Jlumw = Jpcw+Japw

  IntPressure = (0.0+289.76*(Invol-0.01)-3553* (Invol-0.1)^2+28191*(Invol-0.01)^3-1.0767e5*(Invol-0.01)^4+1.5528e5*(Invol-0.01)^5)*pccoef

  Jtotlumgl = Jglpc+Japgl

  Lppc = Lppc*intPressure*pccoef

  Lgn2 = LGng*LumNsat*LumGl2sat

  pccoef = 1

  capGlc = 5+(InsGlc-5)*0.4

  capNc = 150+(InNc-150)*0.6

  lumosm2 = LumGlc*sigGl+LumN*2*SigN

  insosm2 = InNc*2*SigN+InsGlc*sigGl

  JGLUT2 = (LumGl2sat-Cyglu2sat)*VmGlap

  JSGLT1 = Japgl-JGLUT2

  {Globals}

  KmGl1=2

  KmGl2=15

  KmNap=25

  KmGbl=5

  LGg =1

  LGng=0.1

  LNn=1

  VmGlap=1

  VmGlbl=1

  Vpump =1

  PcapGl=0.1

  PcapN=0.1

  Pglpc=0.1

  PNpc= 0.01

  Pprot=0.1

  Lppc=1E-5

  Lpcap= 1E-6

  Lpap=1E-6

  Lpbl=1E-6

  sigGl= 0.2

  SigN=0.5

  Gwcoef=500
