# Supplementary material for: Does apical membrane GLUT2 have a role in intestinal glucose uptake?
Source: F1000Res. 2014 Dec 12;3:304. [Version 1] doi: 10.12688/f1000research.5934.1 (PMC4309173; doi:10.12688/f1000research.5934.1)
Supplement: Peer review history

# Peer review: Referee response for version 1

**DOI:** 10.5256/f1000research.6342.r7456

**Published:** 2015-01-23

**Authors:** Edith Brot-Laroche

**Affiliations:** 1 Centre de recherche des Cordeliers, Université Pierre et Marie Curie, Paris, France

In his manuscript Pr R. Naftalin questions the role of apical GLUT2 in the regulation of intestinal sugar absorption. The manuscript comprises a view of earlier studies on absorption processes and proposes a theoretical model of glucose absorption in the small intestine. Since the main molecular features of sugar transporter in the small intestine have reached consensus, this timely manuscript opens a debate on the regulation of absorption processes including glucose transport, water flow and osmolarity issues. It is an ambitious project to describe a comprehensive model of intestinal sugar absorption considering the many different levels of regulation it involves. However in this manuscript, the kinetics of  transporter expression in and out the enterocyte membranes in the control of absorption processes in sugar absorption were not taken into account in health and disease.

The manuscript starts with a summary of 50 years of research effort in this area. In early days, glucose uptake in enterocytes comprised a Na-coupled electrogenic cotransport system mediated by SGLT1 clone by EM Wright ^1^, and a diffusive component first identified by its resistance to phloridzin inhibition or Na free conditions and later identified as a GLUT family members ^2^^-^^4^. The nature of an epithelial diffusive component was then debated and several mechanisms were proposed including paracellular flow and the presence of facilitated diffusion transport systems in the apical membrane i.e. GLUT5 for fructose and the low affinity transporter GLUT2 for glucose, galactose, fructose and mannose ^2^^, ^^5^. The exit of glucose from enterocytes was described to be mediated by GLUT2 in the basolateral membrane ^5^^-^^7^.

More recent discoveries showed that GLUT2 can also traffic to the apical membrane of enterocytes in humans and rodents ^6^^, ^^7^. GLUT2 trafficking in the apical membrane of epithelial cells has been demonstrated using various techniques including live imaging ^8^^-^^10^. Pr. Naftalin questions the relevance of apical GLUT2 in glucose versus water absorption.

**Major concerns**

1. Pr Naftalin chose to focus on GLUT2-mediated osmoregulation but somehow neglected the dynamics control of sugar transporter densities in the apical and basolateral membranes of enterocytes.  Although the mathematical modeling of this complex system is beyond my expertise, but may I suggest taking the time parameter as a key element in the control sugar absorption.
2. Pr Naftalin reports that apical GLUT2 is not always seen, according to metabolic status of the mice or humans, an assertion that I can share with him. Indeed, apical GLUT2 is visible in animals consuming a sugar diet to increase GLUT2 expression and when glucose is abundant in the lumen. The transient presence of GLUT2 in apical membranes helps thus to understand how the intestine can absorb a large bolus of glucose that overcomes the absorption kinetic capacities of SGLT1. In pathologies altering insulin sensitivity, GLUT2 does not traffic anymore, remaining in apical membranes and increasing intestinal glucose absorption, worsening the status of subjects. In these conditions, apical GLUT2 can be readily observed. This rather simplifies the model of the transcellular transfer of glucose for apical membrane is permanently equipped with the GLUT2 transporter. The model should be amended to take these issues into account, in particular to include the varying levels of GLUT2 in the apical as well as basolateral membranes. This is also true for the SGLT1 transporter.
3. Since transporters are also water channels ^11^^-^^13^ glucose transport will move water in the direction of the glucose gradient through the epithelial layer. Was that feature taken into account in the osmolarity model?
4. Pr. Naftalin identifies conflicting data from my group on facilitative glucose transport, presumably by GLUT2, obtained using brush border membrane vesicles. I would like to give my interpretation of the data published at a time when transporters were characterized only by functional studies. My group identified a second transport system for glucose in addition to Na-cotransport using isolated brush border membrane vesicles, that is, in absence of interference of metabolism. The characteristics of this second system are very close to that identified later on for GLUT2 i.e. a glucose transport system with a low affinity (24 mM) and a high capacity ^14^. This transport is inhibited by cytochalasin B like GLUT2. In the cited papers there is no mention of phloretin inhibition. I suppose that the conflict also come from the nutritional manipulation of guinea pig ^15^ comparing semi-starvation (25% of control food pair feeding experiment) to ad libitum feeding, we showed that the second transport functions was increased in absence of change in the contribution of the cotransport system. At that time, the physiological mechanism underlying the effect of semi-starvation was not analyzed further. Importantly, the effect of starvation was to increase the cotransport of glucose in absence of a contribution of system 2 as expected and confirmed in our studies in mice ^16^. Therefore, it seems that are results are consistent over the different experimental analysis of sugar absorption and the role of GLUT2.
5. The argument that “nonspecific transport of glucose at rates that are correlated with net fluid transport” ^17^ is probably inappropriate since passive diffusion was measured *in vivo *through the urinary recovery of ingested sugars that constitutes an evaluation of passive permeability of the whole intestine including colon. These permeability parameters can hardly be applied as valid for the diffusive component ex vivo in selected part of the small intestine.
6. Different experimental settings for absorption measurements probably cause the discrepancies in the evaluations of transporter K _m_ for GLUT2 and SGLT1. Based on vesicle work ^18^ the Km for the sodium cotransporter and for the facilitative transporter in the BBM of the jejunum is in a ratio of 2 to 25 mM respectively. In addition, studies in rat intestine of Pr Kellet’s group, show cooperativity of the kinetics of glucose transport via GLUT2 ^6^^, ^^19^. Figures in the model should take a better affinity ratio between SGLT1 and GLUT2 mediated transport activities. 

**Minor point:**

GLUT5 is not a glucose transporter in intestine but a fructose transporter. Although it can transport glucose when expressed in Xenopus laevis oocytes.

***Conclusion***

1. The title should include the words “model, mathematics” to stick to the content of the manuscript
2. This manuscript describes the development of a mathematical model of sugar absorption in the intestine, a very challenging project indeed. The main merit is to put forward the many different levels of this complex and multi-compartment mechanism.
3. The model is specially focusing on osmolarity issues in the absorptive process, this may be too far as rapid reading can trouble the reader understanding of the primary function of the transporters, which is primarily glucose transport.
4. A model of this type should include the recent features of sugar absorption and include a kinetic description of events including the varying density of transporters in enterocyte plasma membranes.
5. The role of GLUT2 in pathology should be described to show what are the enhanced and/or decreased elements of the model. Indeed, the absorption phase after a meal should be distinguished from the post absorptive time when the lumen is emptied of glucose.
6. The relative affinities of transporters involved in transepithelial transport need to be corrected to stick to a ratio of affinities measured under the same experimental setting.

I have read this submission. I believe that I have an appropriate level of expertise to confirm that it is of an acceptable scientific standard, however I have significant reservations, as outlined above.

## References

[1] HedigerMACoadyMJIkedaTSWrightEM: Expression cloning and cDNA sequencing of the Na+/glucose co-transporter.*Nature*.1987;330(6146) : 304379-38110.1038/330379a0Reference source2446136

[2] ThorensB: Molecular and cellular physiology of GLUT-2, a high-Km facilitated diffusion glucose transporter. *Int Rev Cytol*.1992;137:209-238142867110.1016/s0074-7696(08)62677-7

[3] JoostHGBellGIBestJDBirnbaumMJCharronMJChenYTDoegeHLodishHFMoleyJFMuecklerMRogersSSchurmannASeinoSThorensB: Nomenclature of the GLUT/SLC2A family of sugar/polyol transport facilitators.*Am J Physiol Endocrinol Metab*.2002;282(4) : 304E974-E97610.1152/ajpendo.00407.2001Reference source11882521

[4] BellGIKayanoTBuseJBBurantCFTakedaJLinDFukumotoHSeinoS: Molecular biology of mammalian glucose transporters.*Diabetes Care*.1990;13(3) :198-208240747510.2337/diacare.13.3.198

[5] CheesemanCI: GLUT2 is the transporter for fructose across the rat intestinal basolateral membrane.*Gastroenterology*.1993;105(4) :1050-1056840584810.1016/0016-5085(93)90948-c

[6] KellettGLBrot-LarocheE: Apical GLUT2: a major pathway of intestinal sugar absorption. *Diabetes*.2005;54(10) : 3043056-306210.2337/diabetes.54.10.3056Reference source16186415

[7] KellettGLBrot-LarocheEMaceOJLeturgueA: Sugar absorption in the intestine: the role of GLUT2.*Annu Rev Nutr*.2008;28: 30435-5410.1146/annurev.nutr.28.061807.155518.Reference source18393659

[8] CohenMKitsbergDTsytkinSShulmanMAroetiBNahmiasY: Live imaging of GLUT2 glucose-dependent trafficking and its inhibition in polarized epithelial cysts. *Open Biol*.2014;4(7) : 30410.1098/rsob.140091Reference source25056286PMC4118605

[9] HelliwellPARichardsonMAffleckJKellettGL: Regulation of GLUT5, GLUT2 and intestinal brush-border fructose absorption by the extracellular signal-regulated kinase, p38 mitogen-activated kinase and phosphatidylinositol 3-kinase intracellular signalling pathways: implications for adaptation to diabetes.*Biochem J*.2000;350(Pt 1) :163-169Reference source10926840PMC1221238

[10] KellettGLHelliwellPA: The diffusive component of intestinal glucose absorption is mediated by the glucose-induced recruitment of GLUT2 to the brush-border membrane. *Biochem J*.2000;350(Pt 1) :155-162Reference source10926839PMC1221237

[11] ZeuthenTMeinildAKLooDDWrightEMKlaerkeDA: Isotonic transport by the Na+-glucose cotransporter SGLT1 from humans and rabbit.*J Physiol*.2001;531(Pt 3) : 304631-64410.1111/j.1469-7793.2001.0631h.xReference source11251046PMC2278509

[12] ZeuthenTZeuthenEMacaulayN: Water transport by GLUT2 expressed in Xenopus laevis oocytes. *J Physiol*.2007;579(Pt 2) : 304345-36110.1113/jphysiol.2006.123380Reference source17158169PMC2075391

[13] LooDDZeuthenTChandyGWrightEM: Cotransport of water by the Na+/glucose cotransporter.*Proc Natl Acad Sci U S A*.1996;93(23) :13367-13370Reference source891759710.1073/pnas.93.23.13367PMC24099

[14] Brot-LarocheESerranoMADelhommeBAlvaradoF: Different temperature sensitivity and cation specificity of two distinct D-glucose/Na+ cotransport systems in the intestinal brush-border membrane. *Ann N Y Acad Sci*.1985;456: 30447-5010.1111/j.1749-6632.1985.tb14843.xReference source3867313

[15] Brot-LarocheEDaoMTAlcaldeAIDelhommeBTriadouNAlvaradoF: Independent modulation by food supply of two distinct sodium-activated D-glucose transport systems in the guinea pig jejunal brush-border membrane.*Proc Natl Acad Sci U S A*.1988;85(17) :6370-6373Reference source341310210.1073/pnas.85.17.6370PMC281973

[16] GouyonFCaillaudLCarriereVKleinCDaletVCitadelleDKellettGLThorensBLeturqueABrot-LarocheE: Simple-sugar meals target GLUT2 at enterocyte apical membranes to improve sugar absorption: a study in GLUT2-null mice. *J Physiol*.2003;552(Pt 3) : 304823-83210.1113/jphysiol.2003.049247Reference source12937289PMC2343460

[17] BijlsmaPBFihnBMSjoqvistAGrootJATaminiauJAJodalM: Water absorption enhances the uptake of mannitol and decreases Cr-EDTA/mannitol permeability ratios in cat small intestine in situ.*Scand J Gastroenterol*.2002;37(7) :799-80612190093

[18] Brot-LarocheESerranoMADelhommeBAlvaradoF: Temperature sensitivity and substrate specificity of two distinct Na+-activated D-glucose transport systems in guinea pig jejunal brush border membrane vesicles.*J Biol Chem*.1986;261(14) :6168-6176Reference source3084480

[19] HelliwellPAKellettGL: he active and passive components of glucose absorption in rat jejunum under low and high perfusion stress. *J Physiol*.2002;544(Pt 2) : 304579-58910.1113/jphysiol.2002.028209Reference source12381828PMC2290612

# Response

**Published:** 2015-01-28

**Authors:** Richard J Naftalin

**Affiliations:** Department of Physiology, King's College London, UK

Dear Prof. Brot Laroche (Edith, EBL)

Thank you for your comments with regard to my commentary about the role of apical GLUT2 in intestinal glucose absorption.     Your comment on the time scale of the changes is an important issue which I did not fully consider in my initial treatment but clearly worth addressing and I will do this now.

The nice diagram (figure 5) in your paper (Ait-Omar et al. 2011) summarizes your view on the dynamics and routes of intestinal glucose absorption.  The paracellular pathway is notably absent from this analysis.  However as I have already mentioned in my answer to GK this is an important route in humans and has relevance to the question of the developing rates of intestinal glucose following acute luminal exposure to glucose during continuous infusion by duodenal gavage (Vanis et al. 2011).  

There are several components with widely differing rates of equilibration relevant to quantitative description of luminal glucose absorption.  First, the rate of delivery to the surface mucosa;  this can be viewed as a rate of change of glucose concentration at a single point, or considered as an integral of the glucose content distributed over the entire absorptive surface.  In considering the latter view, the more distal regions of small intestine will be exposed later than the proximal and to a smaller overall load.  This rarely considered point is treated with simple approximation by Pappenheimer  (Pappenheimer 1998).

Obviously the rate of change of the integral glucose concentration along the entire length of small intestine will be much slower, than the rate as estimated   at the proximal end. 

*Link to figure S1*

  With acute exposure to luminal glucose to 30mM, as modelled in figure S1 A and B luminal glucose concentration is simulated with an exponential rise with a t _½ _≈ 1-2min.  This includes the mixing time within the lumen and diffusion through the unstirred layer to the enterocyte absorptive surface.  Two other major rate processes determine net glucose absorption at the proximal end of the jejunum: the rate of glucose accumulation within the enterocyte cytosol, is difficult to measure in situ, but is assumed to be the main determinant of the rate of glucose absorption. The rate reported in isolated chicken intestinal epithelial cells (Kimmich 1968) has a t _½ _≈ 1-2min.  In vivo this rate likely to be faster   as the force determining this rate is ultimately governed by Na-K ATPase activity which depends on the cell metabolic rate.   The other secondary   rates   determining   cytosolic glucose concentration are mainly via GLUT2 at both apical and baso-lateral membranes and the paracellular pathway.  

Kellett & Helliwell (Kellett & Helliwell 2000) indicate that apical GLUT2 increases as a double exponential,  a small faster component ( t _½ _≈ 1-2min) and a larger slow  component (t _½ _≈ 77 min). I have replaced this double exponential with   a single rate constant (t _½ _= 12 min) to describe the glucose stimulated increase in GLUT2 density at the apical membrane.  The signal for increased apical is initiated when cytosolic glucose concentration >25mM.  This occurs ≈ 1 min after exposure to 30mM luminal glucose.  The simulation shows the effects of three steady state apical GLUT2 densities (0, 1 and 2 nominal units) (Figure 2 A and B). 

In the absence of apical GLUT2 glucose flux via GLUT2 is zero(red lines).  As the density of GLUT2 increases,  following an initial delay, the glucose flux becomes increasing more negative,  reaching a steady state  within ≈ 20 min. This increasing rate of glucose efflux is caused entirely by the slow rise in apical GLUT2 density.  (Lower three lines Figure S1A).  

Of particular note in this simulation are the transient changes in paracellular glucose flux.  As apical GLUT2 density increases, paracellular glucose flux also increases.  This is a consequence of the reduced transcellular glucose flux, which increases the glucose concentration gradient between the lumen and submucosal interstitial fluid; as previously noted (figures 2 and 4). 

The rate constants resulting from the luminal mixing and cytosolic accumulation almost entirely mask any observable glucose influx via GLUT2   occurring during the early phase of intestinal absorption, as envisaged by (Ait-Omar et al 2011) when the cytosol and submucosal glucose < luminal glucose.

EBL “ *Since transporters are also water channels* *^11^* *^-^* *^13^* *glucose transport will move water in the direction of the glucose gradient through the epithelial layer. Was that feature taken into account in the osmolarity model?”*

This question is extremely interesting- to me certainly -(see refs (50,51).  It is evident that water is cotransported via a number of solute cotransporters particularly SGLT1 and GLUT2, but also NKCC(50).  From other model  simulations (not shown) it is apparent that water cotransport  makes no substantial difference to the rates of glucose or Na transport,  but greatly affects glucose and Na-dependent water flows  (not shown). 

Another  noteworthy matter regarding water cotransport concerns the the question  how, when nectarivores  imbibe more than three times their body weight of water per day with little salt content,  they avoid becoming overhydrated.  A solution appears to be that most of the water is retained within the intestinal lumen and excreted via the cloaca rather than kidneys (Karasov & Cork 1994; Caviedes-Vidal et al. 2007; McWhorter et al. 2013). A likely explanation for fluid retention within their gut lumen appears to be that the paracellular glucose diffusion component is maintained at high levels by the by very high metabolic rates and very high rates of splanchnic perfusion.  The  absence of water cotransport by glucose via the paracellular route is likely to be due to its very low reflection coefficient. Thus it is likely that  high paracellular glucose absorption in hummingbirds also prevents glucose from being absorbed via the transcellular route and thereby preventing excessive water absorption.   

 EBL *“The argument that “nonspecific transport of glucose at rates that are correlated with net fluid transport” ^17^ is probably inappropriate since passive diffusion was measured in vivo through the urinary recovery of ingested sugars that constitutes an evaluation of passive permeability of the whole intestine including colon. These permeability parameters can hardly be applied as valid for the diffusive component ex vivo in selected part of the small intestine.”*

This is a question about which I have no expertise.  However, it is probably inaccurate to say that the dual or multi sugar method does not give site-specific gastrointestinal permeability analysis, as has recently been shown (van Wijck et al. 2013).   

EBL *“The relative affinities of transporters involved in transepithelial transport need to be corrected to stick to a ratio of affinities measured under the same experimental setting. ”*

**

In my answer to GK I explained that the assigned transporter parameters within the model relating to affinities are very similar to those obtained with isolated transporters tested in optimal conditions. The apparent operational affinities may differ greatly from the ideal conditions, as the concentrations of transported ligands cannot be held constant in the *trans* compartment(s) when the intestine is operating at near steady state absorption rates.  

EBL *“* *The role of GLUT2 in pathology should be described to show what are the enhanced and/or decreased elements of the model. Indeed, the absorption phase after a meal should be distinguished from the post absorptive time when the lumen is emptied of glucose”.* 

I would like to extend the model to various pathological situations, particularly T2D and hepatic cirrhosis and fatty degeneration and their relationships with obesity.   However this would require extension of the model to include hepatic glucose uptake and circulation (Alexander et al. 2001; Alexander et al. 2002; Li et al. 2003).  However it is evident   from the simple (relatively) analysis outlined already that the local densities of transporters are not the only factor that determines the amplitude of glucose concentration and flow within the splanchnic vascular bed.  Another very important factor which has not been given enough attention is the extent of apical transporter distribution within the intestine and how this affects glucose absorption.  

  

If one assumes that the distribution of SGLT1 and GLUT2 normally covers 30-40% of the available small intestine area  ( 4200 cm ^-2^) (Pappenheimer 1998; Soergel et al. 1968) i.e. 1000-1500 cm ^-2^ then the maximal absorption rate for the entire intestine is approximately 1.0  mmole min ^-1^.   When considered at the level of an individual cell unit, when luminal glucose concentration is high > 30mM,  increased  apical membrane SGLT1densities  will not greatly alter net transcellular absorption,   as this is rate limited by GLUT2 densities in the basolateral membranes and saturation of GLUT2 due to high cytosolic glucose.  Additionally,  as already explained increased apical GLUT2 expression  reduces net transcellular glucose flux   cm ^-2^ of intestine.

   

The observed increase in the area of intestinal SGLT1 distribution with enhanced carbohydrate intake becomes an important factor in total glucose absorption  (Ferraris et al. 1992; Moran et al. 2010). The extent of SGLT1 and GLUT2 distribution within the intestinal   increases both along the crypt-villus axis and along the small intestinal  longitudinal axis   with enrichment of  carbohydrate intake.  As previously discussed apical GLUT2 expression will spread the luminal glucose load over a wider area, so the increased GLUT2 expression along with SGLT1 could lead to higher rates integrated rate of glucose absorption. This as has been noted already may lead to chronic liver damage.  

References

 Ait-Omar, A. et al., 2011. GLUT2 accumulation in enterocyte apical and intracellular membranes: a study in morbidly obese human subjects and ob/ob and high fat-fed mice. *Diabetes*, 60(10), pp.2598–607.  

Alexander, B., Cottam, H. & Naftalin, R., 2001. Hepatic arterial perfusion regulates portal venous flow between hepatic sinusoids and intrahepatic shunts in the normal rat liver in vitro. *Pflügers Archiv : European journal of physiology*, 443(2), pp.257–64.  

Alexander, B., Rogers, C. & Naftalin, R., 2002. Hepatic arterial perfusion decreases intrahepatic shunting and maintains glucose uptake in the rat liver. *Pflügers Archiv : European journal of physiology*, 444(1-2), pp.291–8.  

Caviedes-Vidal, E. et al., 2007. The digestive adaptation of flying vertebrates: high intestinal paracellular absorption compensates for smaller guts. *Proceedings of the National Academy of Sciences of the United States of America*, 104(48), pp.19132–19137.

Ferraris, R.P. et al., 1992. Effect of diet on glucose transporter site density along the intestinal crypt-villus axis. *The American journal of physiology*, 262, pp.G1060–G1068.

Karasov, W.H. & Cork, S.J., 1994. Glucose absorption by a nectarivorous bird: the passive pathway is paramount. *The American journal of physiology*, 267, pp.G18–G26.

Kellett, G.L.& Helliwell, P.A., 2000. glucose-induced recruitment of GLUT2 to the brush-border membrane. *Biochem J*, 162, pp.155–162.

Kimmich, G.A., 1968. Active Sugar Accumulation by Isolated Intestinal Epithelial Cells . *Biochemistry*, 9, pp.3669–3677.

Li, X. et al., 2003. Location and function of intrahepatic shunts in anaesthetised rats. *Gut*, 52(9), pp.1339–46.  

McWhorter, T.J. et al., 2013. Paracellular Absorption Is Relatively Low in the Herbivorous Egyptian Spiny-Tailed Lizard, Uromastyx aegyptia. *PLoS ONE*, 8(4).

Moran, A.W. et al., 2010. Expression of Na+/glucose co-transporter 1 (SGLT1) in the intestine of piglets weaned to different concentrations of dietary carbohydrate. *The British journal of nutrition*, 104(5), pp.647–55.  

Pappenheimer, J.R., 1998. Scaling of dimensions of small intestines in non-ruminant eutherian mammals and its significance for absorptive mechanisms. *Comparative Biochemistry and Physiology - A Molecular and Integrative Physiology*, 121, pp.45–58.

Soergel, K.H., Whalen, G.E. & Harris, J. a, 1968. Passive movement of water and sodium across the human small intestinal mucosa. *Journal of applied physiology (Bethesda, Md. : 1985)*, 24(1), pp.40–48.

Vanis, L. et al., 2011. Effects of small intestinal glucose load on blood pressure, splanchnic blood flow, glycemia, and GLP-1 release in healthy older subjects. *American journal of physiology. Regulatory, integrative and comparative physiology*, 300(5), pp.R1524–R1531.

Van Wijck, K. et al., 2013. Novel multi-sugar assay for site-specific gastrointestinal permeability analysis: A randomized controlled crossover trial. *Clinical Nutrition*, 32(2), pp.245–251.

# Peer review: Referee response for version 1

**DOI:** 10.5256/f1000research.6342.r7045

**Published:** 2015-01-20

**Authors:** Hannelore Daniel

**Affiliations:** 1 Center of Life and Food Sciences, Technical University of Munich, Munich, Germany

This paper by R. Naftalin addresses a facet of gastrointestinal physiology that has for decades caused highly controversial discussions amongst the experts. Carbohydrates in our diet represent the quantitatively most important class of nutrients and a large fraction of our daily caloric intake. After digestion in the upper small intestine glucose derived from starch, glycogen, sucrose and lactose is the nutrient with the highest absorption rate of all dietary components and yet, we still do not understand in details how this is accomplished. There is no doubt that the rheogenic SGLT1 transporter in the brush border membrane is of prime importance and this is known from studies utilizing phloridzin that inhibits SGLT1 with high affinity, from humans suffering from hereditary glucose-galactose malabsorption and from mice lacking a functional SGLT1 protein. One of the critical issues is that SGLT1 when expressed heterologously in a target cell has a very high affinity whereas when glucose absorption is assessed *in vivo* either in rodents or in human intestinal perfusion studies affinities reported are one to two orders of magnitude lower. This discrepancy has been the starting point to search for additional glucose transporters that might be involved. Based on studies in rat intestine G. Kellett finally proposed that at very high luminal glucose concentrations SGLT1 transport function would induce a signalling cascade involving PKC that in turn would cause the recruitment of GLUT2 (a low affinity type uniporter) into the apical membrane from intracellular vesicles. This would allow glucose to move into the cell along a concentration gradient with GLUT2 simultaneously mediating also efflux across the basolateral side as the prime glucose transporting protein constitutively present in this membrane domain. However, as a uniporter GLUT2 could when incorporated into the apical membrane also cause a back-flux of glucose into the intestinal lumen in particular since SGLT1 allows uphill transport of glucose by its rheogenic transport mode. Since all biochemical studies published so far do not give conclusive and convincing data on whether GLUT2 can translocate into the apical membrane and thus contribute to overall glucose absorption, R. Naftalin now takes a modelling approach to assess how feasible a significant contribution of GLUT2 is. Since movement of bulk quantities of solutes (glucose and sodium) by the glucose transporters also induces osmotic gradients and in turn water movement, the conceptual work and the simulations presented also address water movement this as well as blood flow effects. 

   

A model has been build that is plausible and reveals the knowledge base of the author. This model allows changes in a variety of parameters to be visualized for the different compartments when glucose concentrations are increased. Simulations reveal that accumulation of glucose in the cytosol of intestinal epithelial cells mediated by SGLT1 is accompanied by increases in the cytosolic volume whereas an increased density of apical GLUT2 would prevent excessive glucose accumulation at high luminal glucose concentrations by back-flux that would also reduce the enterocyte volume increase. In this context GLUT2 fulfils primarily the role of an osmoregulator in controlling epithelial cell volume that is subject to rapid changes when bulk quantities of the solutes are absorbed. Although the work by R. Naftalin does not prove or disprove that GLUT2 has a important function in overall glucose absorption, it adds some novel ideas and other dimensions to the discussion. GLUT2 and its role in intestinal epithelial cells thus remains a mystery not only in its proposed function in the apical membrane but also for basolateral glucose efflux.

I have read this submission. I believe that I have an appropriate level of expertise to confirm that it is of an acceptable scientific standard.

# Peer review: Referee response for version 1

**DOI:** 10.5256/f1000research.6342.r7370

**Published:** 2015-01-19

**Authors:** George Kellett

**Affiliations:** 1 Department of Biology, University of York, York, UK

**BACKGROUND**

Numerous papers over eight decades since 1935 have reported that intestinal glucose absorption comprises two components, one secondary active and mediated by SGLT1, the other a highly regulated “diffusive” component occurring predominantly at concentrations well above those required to saturate SGLT1. In 1987, Pappenheimer proposed that the “diffusive” component was mediated by SGLT1-dependent solvent-induced paracellular flow through tight junctions, as a result of the concentration of glucose up to ~300 mM in the intercellular spaces ^2^. The field was split by the ensuing debate in which Diamond and Ferraris contested the concept of paracellular flow ^3^^-^^6^. In so doing, they implied that SGLT1 is the only pathway of glucose absorption and denied the existence of two components.

Subsequently, Kellett’s (my) laboratory proposed that the “diffusive” component is mediated by the glucose- and SGLT1-dependent transient insertion of the low affinity basolateral transporter GLUT2 into the apical membrane at high glucose concentrations, so that GLUT2 then mediated a major pathway of transcellular glucose absorption ^7^^-^^9^. Detailed mechanisms for the regulation of apical GLUT2 insertion by diet, sugars, peptides, Ca ^2+^, artificial sweeteners, hormones and obesity have been described ^10^^-^^18^. In effect, the apical GLUT2 model replaced paracellular flow and the impasse in the long-standing debate seemed resolved.

Nevertheless, as cited, some workers have recently reported difficulty detecting apical GLUT2. They therefore continued to deny the existence of any significant pathway other than SGLT1. In fact, the known properties of apical GLUT2 dietary regulation explain both cases. In the report by Gorboulev *et al.* ^19^^,^^20^ mice were starved, which reduces apical GLUT2 and enhances SGLT1; mice also have higher SGLT1 activities than rat. In the report by Roder *et al.* ^21^ using SGLT1 knockout mice, KO and wild-type animals were necessarily maintained on sugar-free diets, which are well known to diminish apical GLUT2 greatly. ^17^

**A NOVEL PARACELLULAR FLOW PROPOSAL**

Professor Naftalin has undertaken the truly daunting task of seeking to provide a comprehensive, integrated model of intestinal glucose absorption. He accepts there are two components of glucose absorption and also that GLUT2 is transiently inserted into the apical membrane. However, in a further twist to the debate, he seeks to reinstate a central role for paracellular flow and thereby reinterpret that of apical GLUT2.

In essence, it is proposed that glucose transport through SGLT1 results in accumulation of cytosolic glucose to supraluminal concentrations in enterocytes. At that point, net absorption of glucose through apical GLUT2 switches to net secretion of accumulated glucose through apical GLUT2 into the lumen; it is then transported across the epithelium by a paracellular channel, along with other luminal glucose. Apical GLUT2 therefore acts, not as a direct transcellular transporter, but as a shunt to supply a major paracellular pathway of transepithelial absorption.

**Paracellular flow**

The proposal depends absolutely on the view that paracellular flow of glucose through tight junctions exists and, furthermore, that it occurs with rates comparable to observed luminal disappearance in perfusions in vivo. However, the concept of paracellular flow advanced by Pappenheimer lost favour during the debate with Diamond and Ferraris ^22^. In particular, they argued convincingly that paracellular markers, if they cross the intestinal barrier at all, do so only in low amounts after experiments of many hours or even longer, when the integrity of tissue becomes questionable. Even in cited example of cat, reported paracellular flow of glucose is only ~10% of glucose absorption. Thus the rate of absorption of paracellular markers is minimal to negligible compared with that for glucose.

Nevertheless, because of its potential importance, my laboratory made strenuous attempts to detect paracellular flow ^12^. Mannitol, the “gold standard” of paracellular markers was used, since L-glucose has a low affinity for SGLT1. Rats were maintained on a high carbohydrate diet and food was flushed from jejunum just before perfusion *in vivo*; the perfusion was single pass at low flow rate and net glucose absorption (total absorption minus secretion) was determined chemically as luminal disappearance. At 75 mM luminal glucose, the steady-state rate of glucose absorption was established within just 15 min and was some 200-fold greater than for mannitol; see Table 1 of ref 12. “Closing” tight junctions by inhibiting myosin phosphorylation had no effect on mannitol clearance; nor did clearance change over a wide range of glucose concentrations at constant osmolarity (balanced with mannitol to 75 mM total sugar). Changes in water absorption correlated with changes in rapidly inserted apical GLUT2, not mannitol clearance.

Studies from other groups lead to same conclusion. In the work of Brot-Laroche and colleagues on apical GLUT2, fructose transport studies were done in vesicles ^17^; paracellular flow could not be involved.  Apical GLUT2 mediated 60% of fructose transport, the rest was by GLUT5. The results with GLUT2 knockout mice correlated well with fructose and glucose perfusion data, one could be predicted from the other. Since GLUT5 and GLUT2 are facilitative transporters, there can be no concentration of the transcellular gradient *in vivo*, yet fructose absorption at high luminal concentrations *in vivo *shows the same apparent linearity as glucose ^2^.

Resistin ^23^ and metformin ^24^ promote AMP-mediated rapid insertion of apical GLUT2 in mice and rat to result in an increase in associated glucose absorption; there is no effect on minimal mannitol clearance. Notably, SGLT1 membrane density and ∆Isc were halved. This switch away from SGLT1 at high glucose concentrations was first observed in a study with the powerful AMP-kinase activator, AICAR, which induces a 3-fold increase in glucose absorption through a comparable increase in apical GLUT2 ^25^. Simultaneously, SGLT1 is degraded to such an extent as to be almost undetectable (within ~30 min), so that only GLUT2 is at the apical membrane. The switch to the facilitative, energy-independent apical GLUT2 may represent a response to energy stress as the energy-dependent SGLT1 reaches maximal capacity.

Reports that paracellular flow is minimal to negligible under the conditions of apical GLUT2 studies have not been cited. Nor have any new experimental data been presented to change the debate on paracellular flow. The integrity of the intestinal barrier is paramount, as immunologists surely agree.

**Intestinal transport is simulated with a computer-based model**

The novel paracellular flow proposal is based entirely on computer simulation of what is described as a “multiplicity and complexity of interactive processes”. As the extensive mathematical appendix so lucidly shows, simulation depends on many steps and their interactions, the choice of many parameters and their values, the concentrations of glucose and Na ^+^ at specific places within various compartments, and so on. Inevitably, a serious issue is that some, possibly most, values are of great uncertainty or simply unknown.

Two key examples suffice to make the point. The K _t_ of glucose for SGLT1 is taken as 17 mM, in contrast to reports of 23 ^26^ and 26 mM ^7^ *in vivo*. The K _t_ of GLUT2 is also taken as 17 mM, reported for expression in oocytes. In contrast, the  value of 56 ± 14 mM, based on an empirical sigmoid curve analysis of *in vivo* data ^7^, is described as “very high”, implying the “diffusive” component is not GLUT2 but paracellular flow. In fact, the K _t_ of GLUT2 for uptake is 48 ± 5 mM for basolateral membrane vesicle preparations ^27^. Moreover, the question of asymmetry in this bidirectional transporter seems not to have been addressed: V _max_ for uptake is ~6-fold greater than for efflux ^27^. Such differences and uncertainties for most parameters surely have a considerable impact on simulation. Indeed, simply taking the K _t _values of SGLT1 and GLUT2 to be the same as each other and much lower than the literature consensus strongly biases simulation outcome.

**Transcellular Glucose Gradients *In Vivo***

It is widely accepted that secondary active transport by SGLT1 results in glucose accumulation to enterocyte concentrations greater than in the lumen of intestinal preparations *in vitro*. This is because the K _t_ for SGLT1 is very much lower than *in vivo* (sub mM v 26 mM) and because preparations such as everted sacs have poor clearance. The question is whether glucose accumulation occurs *in vivo*. If, as the balance of evidence indicates, there is negligible paracellular flow, then the transcellular gradient must be downhill (l-m-s) when luminal concentrations are high. Other evidence supporting this conclusion has been reviewed ^3^^-^^5^.

**Recycling of Glucose and Na ^+^ Across Intestine**

Recycling of certain key nutrients across the intestine is vital to its function. Thus Na ^+^ is recycled paracellularly to the lumen at high rates through claudin-15, to support continued activity of SGLT1 and other Na ^+^-dependent transporters ^28^. Knockout of claudin-15 results in abnormally low luminal Na ^+^ (~8 mM v ~57 mM for wild-type) and a large impairment in glucose absorption (∆Isc). Recycling of Na ^+^ is important when luminal glucose concentrations are low, e.g. between meals or overnight, in starvation and desquamation. Thus SGLT1 is the only transporter capable of driving glucose uphill against its gradient to plasma and therefore preventing loss of glucose by secretion through apical GLUT2.

Conversely, glucose is important for Na ^+^ recovery.  A clear example is streptozocin-diabetes, which is characterised by hyperglycaemia and hyponatraemia; a permanently high level of apical GLUT2 is also found in diabetic rats ^29^. When luminal glucose is less than in plasma, secretion through apical GLUT2 ^16^ is three-fold greater in diabetic than in normal rats and is increased by phloridzin ^30^. Thus recycling of glucose through apical GLUT2 is associated with recovery of Na ^+^ through SGLT1, providing potential compensation for loss of Na ^+^ by urinary excretion in diabetes. SGLT1 will also play a role in recovery of water secreted by apical GLUT2.

In the scenarios just outlined, there is clear physiological advantage to secretion of either Na ^+^ or glucose in their mutual recovery at low luminal concentrations. At high concentrations in the absence of paracellular flow, the switch to facilitated transcellular absorption through apical GLUT2 would be energy efficient and, as noted for periods of energy stress, might even be accompanied by a reduction of energy-dependent SGLT1. All that is required is a natural reversal of the transcellular concentration gradient in response to a fresh luminal glucose load. Energy-linked recycling of metabolic substrates used to be thought of as a waste of energy, a so-called “futile cycle.” It is now recognised as an amplification mechanism for rapid mobilisation of fresh substrate, or, by analogy, uptake of incoming glucose when absorption and secretion are initially comparable at low luminal glucose concentrations.

In addition to its roles as a transporter and in the mutual recovery of Na ^+^ and glucose, SGLT1 plays a major role in regulating apical GLUT2.

**SUMMARY**

1. This paper highlights the sheer complexity of developing an integrated model of intestinal glucose absorption. Such contributions to the literature are rare; they are valuable for forcing us to think about many things that we have yet to learn about, including apical GLUT2 and the *in vivo* situation, and to promote debate. Significant issues are inevitable, though, when such an ambitious objective is in mind.
2. The novel model places paracellular flow at the heart of intestinal glucose absorption. However, new experimental evidence necessary to justify such a role is not presented. The account of the debate on paracellular flow is very selective and gives the erroneous impression that the concept is widely accepted. At best, it is controversial. A fuller account should be given.
3. Specifically, recent papers from several groups report that paracellular flow is minimal under the conditions of apical GLUT2 studies, when large changes in absorption, apical GLUT2 and even SGLT1 are seen. Reinterpretation of apical GLUT2 data to support the novel paracellular flow model is not possible.
4. Simulation results are presented for just one luminal glucose concentration (50 mM) and two rather selective values of K _m_ for key transporters; there is no consideration of GLUT2 asymmetry. A much wider range of factors, concentrations and parameter values should be explored, including the absence of paracellular flow.
5. There is significant literature evidence to support the view that, when the glucose concentration in the lumen is much higher than in plasma, the transcellular glucose gradient (l-m-s) is downhill all the way *in vivo*. The differences between *in vivo *and *in vitro *situations should be clarified.
6. Emphasis on recycling of glucose through apical GLUT2 has been as a shunt to serve paracellular flow. Evidence that recycling of glucose and Na ^+^ through SGLT1 is vital to intestinal function should be included.
7. New title? “Computer simulation of an integrative model of intestinal glucose absorption”

I have read this submission. I believe that I have an appropriate level of expertise to confirm that it is of an acceptable scientific standard, however I have significant reservations, as outlined above.

## References

[1] DonhofferS: Über die elektive resorption der zucker. *Arch Exp Pathol u Pharmakol*.1935;177:689-692

[2] PappenheimerJRReissKZ: Contribution of solvent drag through intercellular junctions to absorption of nutrients by the small intestine of the rat.*J Membr Biol*.1987;100(2) :123-136343056910.1007/BF02209145

[3] KellettGL: The facilitated component of intestinal glucose absorption.*J Physiol*.2001;531(Pt 3) : 304585-59510.1111/j.1469-7793.2001.0585h.xReference source11251042PMC2278489

[4] KellettGLBrot-LarocheE: Apical GLUT2: a major pathway of intestinal sugar absorption. *Diabetes*.2005;54(10) : 3043056-306210.2337/diabetes.54.10.3056Reference source16186415

[5] KellettGLBrot-LarocheEMaceOJLeturgueA: Sugar absorption in the intestine: the role of GLUT2.*Annu Rev Nutr*.2008;28: 30435-5410.1146/annurev.nutr.28.061807.155518.Reference source18393659

[6] KelletGL: Alternative perspective on intestinal calcium absorption: proposed complementary actions of Ca(v)1.3 and TRPV6.*Nutr Rev*.2011;69(7) : 304347-37010.1111/j.1753-4887.2011.00395.x21729089

[7] KellettGLHelliwellPA: The diffusive component of intestinal glucose absorption is mediated by the glucose-induced recruitment of GLUT2 to the brush-border membrane. *Biochem J*.2000;15(Pt 1) :155-162Reference source10926839PMC1221237

[8] HelliwellPARichardsonMAffleckJKellettGL: Stimulation of fructose transport across the intestinal brush-border membrane by PMA is mediated by GLUT2 and dynamically regulated by protein kinase C.*Biochem J*.2000;350(Pt 1) :149-154Reference source10926838PMC1221236

[9] HelliwellPARichardsonMAffleckJKellettGL: Regulation of GLUT5, GLUT2 and intestinal brush-border fructose absorption by the extracellular signal-regulated kinase, p38 mitogen-activated kinase and phosphatidylinositol 3-kinase intracellular signalling pathways: implications for adaptation to diabetes.*Biochem J*.2000;350(Pt 1) :163-169Reference source10926840PMC1221238

[10] HelliwellPAKelletGL: The active and passive components of glucose absorption in rat jejunum under low and high perfusion stress. *J Physiol*.2002;544(Pt 2) : 304579-58910.1113/jphysiol.2002.028209Reference source12381828PMC2290612

[11] HelliwellPARumsbyMGKelletGL: Intestinal sugar absorption is regulated by phosphorylation and turnover of protein kinase C betaII mediated by phosphatidylinositol 3-kinase- and mammalian target of rapamycin-dependent pathways. *J Biol Chem*.2003;278(31) : 30428644-2865010.1074/jbc.M301479200Reference source12766174

[12] MaceOJMorganELAffleckJAListerNKellettGL: Calcium absorption by Cav1.3 induces terminal web myosin II phosphorylation and apical GLUT2 insertion in rat intestine. *J Physiol*.2007;580(Pt 2) : 304605-61610.1113/jphysiol.2006.124784Reference source17272349PMC2075544

[13] MorganELMaceOJAffleckJKellettGL: Apical GLUT2 and Cav1.3: regulation of rat intestinal glucose and calcium absorption. *J Physiol*.2007;580(Pt 2) : 304593-60410.1113/jphysiol.2006.124768Reference source17272350PMC2075547

[14] MaceOJAffleckJPatelNKellettGL: Sweet taste receptors in rat small intestine stimulate glucose absorption through apical GLUT2. *J Physiol*.2007;582(Pt 1) : 304379-39210.1113/jphysiol.2007.130906Reference source17495045PMC2075289

[15] MaceOJListerNMorganEShepherdEAffleckJHelliwellPBronkJRKellettGLMeredithDBoydRPieriMBaileyPDPettcrewRFoleyD: An energy supply network of nutrient absorption coordinated by calcium and T1R taste receptors in rat small intestine. *J Physiol*.2009;587(Pt 1) : 304195-21010.1113/jphysiol.2008.159616Reference source19001049PMC2670033

[16] Ait-OmarAMonteriro-SupulvedaMPoitouCLe GallMCotillardAGiletJGarbinKHoullierAChateauDLacombeAVeyrieNHugolDTordjmanJMagnanCSerradasPClémentKLeturgueABrot-LarocheE: GLUT2 accumulation in enterocyte apical and intracellular membranes: a study in morbidly obese human subjects and ob/ob and high fat-fed mice. *Diabetes*.2011;60(10) : 3042598-260710.2337/db10-1740Reference source21852673PMC3178286

[17] GouyonFCaillaudLCarriereVKleinCDaletVCitadelleDKellettGLThorensBLeturgueABrot-LarocheE: Simple-sugar meals target GLUT2 at enterocyte apical membranes to improve sugar absorption: A study in GLUT2-null mice. *J Physiol*.2003;552(Pt 3) : 304823-83210.1113/jphysiol.2003.049247Reference source12937289PMC2343460

[18] TobinVLe GallMFioramontiXStolarczykEBlazguezAGKleinCPrigentMSerradasPCuifMHMagnanCLeturgueABrot-LarocheE: Insulin internalizes GLUT2 in the enterocytes of healthy but not insulin-resistant mice. *Diabetes*.2008;57(3) : 304555-56210.2337/db07-0928Reference source18057092

[19] GorboulevVSchurmannAVallonVKippHJaschkeAKlessenDFriedrichAScherneckSRiegTCunardRVeyhl-WichmannMSrinivasanABalenDBreljakDRexhepajRParkerHEGribbleFMReimannFLangFWieseSSabolicISendtnerMKoepsellH: Na+-D-glucose Cotransporter SGLT1 is Pivotal for Intestinal Glucose Absorption and Glucose-Dependent Incretin Secretion.*Diabetes*.2012;61(1) : 304187-19610.2337/db11-1029Reference source22124465PMC3237647

[20] KellettGL: Comment on: Gorboulev et al. Na+-D-glucose cotransporter SGLT1 Is pivotal for intestinal glucose absorption and glucose-dependent incretin secretion. *Diabetes*.2012;61(6) : 304e410.2337/db11-1793Reference source22124465PMC3237647

[21] RoderPVGeillingerKEZietekTSThorensBKoepsellHDanielH: The role of SGLT1 and GLUT2 in intestinal glucose transport and sensing. *PLoS One*.2014;9(2) : 304e8997710.1371/journal.pone.0089977Reference source24587162PMC3935955

[22] FerrarisRPDiamondJ: Regulation of intestinal sugar transport. *Physiol Rev*.1997;77(1) :257-302Reference source901630410.1152/physrev.1997.77.1.257

[23] KrimiRBLetteronPChedidPNazaretCDucrocRMarieJC: Resistin-like molecule-beta inhibits SGLT-1 activity and enhances GLUT2-dependent jejunal glucose transport. *Diabetes*.2009;58(9) : 3042032-203810.2337/db08-1786Reference source19502416PMC2731541

[24] SakarYMeddahBFaouziMACherrahYBadoADucrocR: Metformin-induced regulation of the intestinal D-glucose transporters. *J Physiol Pharmacol*.2010;61(3) :301-307Reference source20610860

[25] WalkerJJijonHBDiazHSalehiPChurchillTMadsenKL: 5-aminoimidazole-4-carboxamide riboside (AICAR) enhances GLUT2-dependent jejunal glucose transport: a possible role for AMPK. *Biochem J*.2005;385(Pt 2) : 304485-49110.1042/BJ20040694Reference source15367103PMC1134720

[26] DebnamESLevinRJ: An experimental method of identifying and quantifying the active transfer electrogenic component from the diffusive component during sugar absorption measured in vivo. *J Physiol*.1975;246(1) : 304181-19610.1113/jphysiol.1975.sp010885Reference source1133782PMC1309409

[27] Please write authors as SurnameXSurnameY: The Na+-independent D-glucose transporter in the enterocyte basolateral membrane: orientation and cytochalasin B binding characteristics. *J Membr Biol*.1987;97(3) :259-266362575910.1007/BF01869228

[28] TamuraAHayashiHImasatoMYamazakiYHagiwaraAWadaMNodaTWatanabeMSuzukiYTsukitaS: Loss of claudin-15, but not claudin-2, causes Na+ deficiency and glucose malabsorption in mouse small intestine. *Gastroenterology*.2011;140(3) : 304913-92310.1053/j.gastro.2010.08.006Reference source20727355

[29] CorpeCPBasalehMMAffleckJGouldGJessTJKellettGL: The regulation of GLUT5 and GLUT2 activity in the adaptation of intestinal brush-border fructose transport in diabetes. *Pflugers Arch*.1996;432(2) :192-201866229410.1007/s004240050124

[30] LevineGMShiauYFDerenJA: Characteristics of intestinal glucose secretion in normal and diabetic rats. *Am J Physiol*.1982;242(5) :G455-G459Reference source621110110.1152/ajpgi.1982.242.5.G455

# Response

**Published:** 2015-01-21

**Authors:** Richard J Naftalin

**Affiliations:** Department of Physiology, King's College London, UK

Dear Professor Kellett (George, GK),

Thank you for reviewing my commentary on the role of apical GLUT2 in intestinal absorption of glucose, whose main focus is examining quantitatively how apical GLUT2 interactions affect intestinal glucose transport.  Your  research has generated much interest and controversy over the last fifteen years.    I am fairly convinced that the problem that it has resurrected – namely, coping strategies with excessive glucose absorption and its subsequent accumulation within the splanchnic circulation, has important implications for the pathophysiology of glucose absorption and metabolism.   The spin offs implicating the  role of gut incretins, autocoids and hormones in control of glucose appetite, taste and absorption with ramifications in such topics as obesity and type II diabetes are likely to be of critical importance and subject of  research for the foreseeable future.    Nevertheless, I am in substantial disagreement with some of the topics you raise.     Some of the points I will make refer to simple clarifications and others are more fundamental. 

 Point 1: You GK say “ *At that point, net absorption of glucose through apical GLUT2 switches to net secretion of accumulated glucose through apical GLUT2 into the lumen; it is then transported across the epithelium by a paracellular channel, along with other luminal glucose. Apical GLUT2 therefore acts, not as a direct transcellular transporter, but as a shunt to supply a major paracellular pathway of transepithelial absorption”.*

Possibly, there is a slight misreading here.  GLUT2 does indeed function as an apical membrane shunt facilitating glucose reflux, thereby reducing cytosolic glucose concentration.  This has the secondary effect of reducing net glucose flux across the baso-lateral membrane, thus reducing   interstitial glucose concentration and thereby increasing the concentration gradient between the lumen and interstitial fluids.  This increased concentration gradient generates the increased diffusive flux via the parallel paracellular pathway.    No adjustment of paracellular parameters is required to accommodate this increased paracellular flow.  The mere fact that a paracellular route is present in parallel with the transcellular route will generate such an increase in flow if transcellular flux is reduced inhibited. 

Point 2:  GK contends that the (Ferraris & Diamond 1997) review eliminates the possibility of a significant functional paracellular route for glucose absorption - as you say *“Diamond and Ferraris contested the concept of paracellular flow ^3-6^….. In so doing, they implied that SGLT1 is the only pathway of glucose absorption and denied the existence of two components” *and later you say *“the apical GLUT2 model replaced paracellular flow and the impasse in the long-standing debate seemed resolved*”.  Perhaps!  But, then again maybe not – nothing much in Physiology is wholly resolved.   

The implication is that Ferraris and Diamond, refuted the functional existence of the paracellular absorptive pathway is contradicted by substantial literature in which the in vivo technique of monitoring passive intestinal permeability using dual sugar absorption technique e.g. lactulose and rhamnose (Laker & Menzies 1977; Bjarnason et al. 1995) demonstrates a considerable element of passive intestinal absorption of  un-metabolized  sugars in humans in vivo.      Furthermore, it is evident that neither Diamond nor his erstwhile colleagues, Ferraris and Karasov, any longer hold fast to the view that paracellular sugar fluxes are a negligible component of intestinal absorption.       Passive paracellular glucose absorption is now thought to be the dominant route of absorption in nectarivores and be of major importance in several reptiles, rodents and mammals.  It is apparent that there is wide species variability in passive intestinal absorption of sugars: from highest  in the American robin with 92% passive (paracellular) absorption  to lowest, 2.1 % in rabbits (Karasov & Cork 1994; Secor et al. 1994; Ferraris 2001).  Humans have around 20% passive absorption, assuming they have not been drinking alcohol excessively, or not  treated with non-steroidal anti-inflammatory drugs, when the paracellular flow increases by at least two-fold (Bjarnason et al. 1984).   Thus, I do not agree with your assertion that paracellular glucose transport is generally negligible – this is particularly not the case when luminal glucose concentrations exceed 25mM.    The low mannitol “clearance” you refer to should be compared with the passive, rather than the active component of glucose absorption. Passive glucose and galactose loss from rat jejunum in the presence of 0.5mM phloridzin does not differ significantly from sorbose - which is poorly selective for GLUTs and SGLTs (Debnam & Levin 1975). 

 Point 3:  A clarification:  the assigned Kt of glucose for SGLT1 transport is 2mM and for Na is 25mM and for GLUT2 at both apical and basolateral membranes the Kt is 17mM.  This requires clarification and I will amend the appendix and include these values in the legends to Figure 1. 

These values give an “operational” Kt for net glucose flux via SGLT1, as obtained from the model     ≈17mM Figure 3A. The flux via apical GLUT2 with an absent paracellular component Figure 3B has an “operational” Kt   around 25mM. These values are well within the observed values reported. 

For those   unfamiliar with modelling complex processes,    the operational Kt’s are not necessarily the same as parameters assigned to the transporter.  The operational Kt for net transport is a lumped parameter that depends upon a large number of interactions.  Operational   “parameters” –are actually variables and only equal to the assigned parameters in the ideal *zero-trans* condition i.e. when the opposing side of the transporter contains zero ligand and back flux is zero.   Whilst this condition may hold in a few in vitro conditions, such as uptake into oocytes, or exit fluxes from cells or vesicles into large volume of bathing solution, it is unattainable with net intestinal transport, where the cytosolic and interstitial fluids contain transported ligands at substantial concentrations.

Using pharmacological tools in complex whole tissue preparations to dissect the parameters of individual transport process is likely to be a crude and sometimes misleading practice.     However, adoption of more precise reductive approaches does not circumvent the problem of assessing how individual elements function within the integrated whole organ as is being examined here.  This is the role of modelling and simulation which depends of course on use of experimental data.    Apropos   I am disappointed that GK has made no comment on the action of phloretin as he attributed to it the unique effect of inhibiting apical GLUT2(Kellett & Helliwell 2000).  In addition to the other reported effects of phloretin I have cited   (Wright et al. 2011) have mentioned that phloretin also inhibits SGLT1 (Ki 50 µM). They  infer that Kellett and Helliwell underestimated the role of SGLT1 in intestinal transport.   

Point 4:  GK contends *that the simulation results are presented for just one luminal glucose concentration (50 mM) and two rather selective values of K* *_m_* *for key transporters; there is no consideration of GLUT2 asymmetry. A much wider range of factors, concentrations and parameter values should be explored, including the absence of paracellular flow.*

 It is incorrect to imply that the results are presented at just one luminal glucose concentration.  This is true only for figure 1.  In figures 2 and 3, luminal glucose concentration is varied between 0 and 50 mM and the steady state transepithelial glucose transport rates are simulated in different conditions (see figure 3A and 4A). In figure 4, the luminal glucose concentration is held at 30mM to simulate the conditions observed by (Kellett & Helliwell 2000). With phloridzin present it is certainly correct that the luminal> cytosolic > interstitial glucose concentration.  However   when phloretin is added in addition to simulate the Kellett-Helliwell experiment and it is assumed   only to inhibit apical GLUT2, no significant change in transepithelial glucose flux is observed.  The reason for this is that the reduced transcellular flow is compensated by enhanced paracellular flow.  Only when phloretin is assumed to block paracellular flow in addition to transcellular flow is any inhibitory effect of phloretin on transepithelial glucose transport observed.   

Many other conditions could be simulated e.g. varying Na concentrations, varying osmolarity etc; however the main point of this review is to illustrate some of the fallacies relating to apical GLUT2 function without excessive overload.   

Point 5 GK: *There is no consideration  of GLUT2 asymmetry*… (Maenz & Cheeseman 1987) reported that the kinetic parameters observed in baso-lateral membrane vesicles from rat small intestine.  They found that the Kt for uptake was 48±5 mM glucose, whereas for influx it was 23±2mM.  A later review reported that GLUT2 in isolated hepatocytes has a Km of approximately 20mM and is symmetrical  (Thorens 1993).    I will add a reference to Thorens review on GLUT2. 

Point 6 *.  *GK makes the point that *“Emphasis on recycling of glucose through apical GLUT2 has been as a shunt to serve paracellular flow. Evidence that recycling of glucose and Na* *^+^* *through SGLT1 is vital to intestinal function should be included*.”

I take this to mean glucose and Na recovery from the lumen via SGLT e.g. during periods of starvation e.g. at night.  It is certainly likely that glucose may leak via the transcellular route when the lumen is glucose free. This may also occur via the paracellular route and recovery via SGLT is an important function.  This point was made very well by (Boyd & Parsons 1976) and says much about SGLT capacity to recapture glucose leaked via either trans or paracellular routes from the blood but no much about the specific role of apical GLUT2. However it is not  very relevant to the main topic of debate here,  which is the mode of intestinal transport with high luminal glucose concentrations. 

Point 7. *GK suggests a  new title  “Computer simulation of an integrative model of intestinal glucose absorption”*

 I do not feel that a changed title is a warranted.  I agree that computer simulation of net glucose transport lies at the heart of this commentary,   however this is used simply as a tool-  a means  to illustrate the cardinal point  that GLUT2 does not have a functional role in augmenting net glucose transport *in the way that GK has suggested*  - namely during luminal overload *. * However by its possible alternative roles in spreading the luminal glucose load over a wider absorptive area  and also preventing osmotic stress,  GLUT2 induction may augment net glucose transport,  as has been shown. 

**References**

Bjarnason, I., MacPherson, A. & Hollander, D., 1995. Intestinal permeability: An overview. *Gastroenterology*, 108, pp.1566–1581.

Bjarnason, I., Peters, T.J. & Wise, R.J., 1984. The leaky gut of alcoholism: possible route of entry for toxic compounds. *Lancet*, 1(January), pp.179–182.

Boyd, C. & Parsons, D., 1976. Effects of vascular perfusion on the accumulation distribution and transfer of 3-O-methyl-D-glucose within and across the small intestine. *J Physiol*, 274, pp.17–36.

Debnam, B.Y.E.S. & Levin, R.J., 1975. AN EXPERIMENTAL METHOD OF IDENTIFYING AND QUANTIFYING THE ACTIVE TRANSFER ELECTROGENIC COMPONENT FROM THE DIFFUSIVE COMPONENT DURING SUGAR ABSORPTION MEASURED IN VIVO From the Department of Physiology , University of Sheffield , side have been measured in. *J Physiol*, pp.181–196.

Ferraris, R. & Diamond, J., 1997. Regulation of intestinal sugar transport. *Physiological Reviews*, 77(1), pp.257–302. Available at: http://physrev.physiology.org/content/77/1/257.short.

Ferraris, R.P., 2001. Dietary and developmental regulation of intestinal sugar transport. *The Biochemical journal*, 360, pp.265–276.

Karasov, W.H. & Cork, S.J., 1994. Glucose absorption by a nectarivorous bird: the passive pathway is paramount. *The American journal of physiology*, 267, pp.G18–G26.

Kellett, G.L. & Helliwell, P.A., 2000. glucose-induced recruitment of GLUT2 to the brush-border membrane. *Biochem J*, 162, pp.155–162.

Laker, M. & Menzies, I., 1977. Increase in human intestinal permeability following ingestion of hypertonic solutions. *J Physiol*, 265, pp.881–894.

Maenz, D.D. & Cheeseman, C.I., 1987. The Na+-independent d-glucose transporter in the enterocyte basolateral membrane: Orientation and cytochalasin B binding characteristics. *The Journal of Membrane Biology*, 97, pp.259–266.

Secor, S.M., Stein, E.D. & Diamond, J., 1994. Rapid upregulation of snake intestine in response to feeding: a new model of intestinal adaptation. *The American journal of physiology*, 266, pp.G695–G705.

Thorens, B., 1993. Facilitated Glucose Transporters in epithelial cells. *Annu Review Physiol.*, pp.591–608.

Wright, E.M., Loo, D.D.F. & Hirayama, B.A., 2011. Biology of human sodium glucose transporters. *Physiological reviews*, 91(2), pp.733–794. Available at: http://www.ncbi.nlm.nih.gov/pubmed/21527736 [Accessed September 10, 2014].
